# GRK2 blockade with βARKct is essential for cardiac β_2_-adrenergic receptor signaling towards increased contractility

**DOI:** 10.1186/1478-811X-11-64

**Published:** 2013-08-28

**Authors:** Norma C Salazar, Ximena Vallejos, Ashley Siryk, Giuseppe Rengo, Alessandro Cannavo, Daniela Liccardo, Claudio De Lucia, Erhe Gao, Dario Leosco, Walter J Koch, Anastasios Lymperopoulos

**Affiliations:** 1 Department of Pharmaceutical Sciences, Laboratory for the Study of Neurohormonal Control of the Circulation, Nova Southeastern University College of Pharmacy, Fort Lauderdale, FL 33328, USA; 2 Division of Cardiology, Department of Internal Medicine, Cardiovascular Sciences and Immunology, University “Federico II”, Naples, Italy; 3 Center for Translational Medicine, Temple University, Philadelphia, PA 19140, USA

**Keywords:** Cardiac β_2_–adrenergic receptor, Pro-contractile signaling, Post-myocardial infarction survival, GRK2 inhibition, GRK5

## Abstract

**Background:**

β_1_- and β_2_–adrenergic receptors (ARs) play distinct roles in the heart, e.g. β_1_AR is pro-contractile and pro-apoptotic but β_2_AR anti-apoptotic and only weakly pro-contractile. G protein coupled receptor kinase (GRK)-2 desensitizes and opposes βAR pro-contractile signaling by phosphorylating the receptor and inducing beta-arrestin (βarr) binding. We posited herein that GRK2 blockade might enhance the pro-contractile signaling of the β_2_AR subtype in the heart. We tested the effects of cardiac-targeted GRK2 inhibition in vivo exclusively on β_2_AR signaling under normal conditions and in heart failure (HF).

**Results:**

We crossed β_1_AR knockout (B1KO) mice with cardiac-specific transgenic mice expressing the βARKct, a known GRK2 inhibitor, and studied the offspring under normal conditions and in post-myocardial infarction (MI). βARKct expression in vivo proved essential for β_2_AR-dependent contractile function, as β_2_AR stimulation with isoproterenol fails to increase contractility in either healthy or post-MI B1KO mice and it only does so in the presence of βARKct. The main underlying mechanism for this is blockade of the interaction of phosphodiesterase (PDE) type 4D with the cardiac β_2_AR, which is normally mediated by the actions of GRK2 and βarrs on the receptor. The molecular “brake” that PDE4D poses on β_2_AR signaling to contractility stimulation is thus “released”. Regarding the other beneficial functions of cardiac β_2_AR, βARKct increased overall survival of the post-MI B1KO mice progressing to HF, via a decrease in cardiac apoptosis and an increase in wound healing-associated inflammation early (at 24 hrs) post-MI. However, these effects disappear by 4 weeks post-MI, and, in their place, upregulation of the other major GRK in the heart, GRK5, is observed.

**Conclusions:**

GRK2 inhibition in vivo with βARKct is absolutely essential for cardiac β_2_AR pro-contractile signaling and function. In addition, β_2_AR anti-apoptotic signaling in post-MI HF is augmented by βARKct, although this effect is short-lived.

## Background

Despite recent advances in prevention and management of heart disease, death due to post-myocardial infarction (MI) heart failure (HF) continues to rise and new treatments are needed [1]. β_1_- and β_2_–adrenergic receptors (ARs) are the main stimulatory receptors of cardiac function but are now known to play clearly distinct roles in cardiac physiology and pathology [2-5]. For instance, cardiomyocyte contraction is readily stimulated by β_1_ARs but not β_2_ARs and β_1_AR signaling is generally considered pro-apoptotic whereas β_2_AR signaling anti-apoptotic in the heart [2-5]. These differences might be explained by differences in the signaling complexes assembled by activation of these two βARs: β_1_AR forms a complex with phosphodiesterase (PDE) 4D8 directly when inactive, and agonist binding dissociates it [6,7]. Additionally, β_1_AR does not readily bind the receptor adapter proteins beta-arrestins (βarrs) following its agonist-promoted phosphorylation by G protein coupled receptor kinases (GRKs), the most prominent members of which in the heart are GRK2 and −5 [8-11]. In contrast, β_2_AR recruits another PDE variant upon its agonist activation, PDE4D5, via its interaction with βarrs following its GRK-dependent phosphorylation [6,7,12-16]. PDE recruitment to the receptor’s signaling complex plays a crucial role in compartmentalizing the cyclic adenosine monophosphate (cAMP) signal and thereby tightly regulating βAR-stimulated contractility [7]. It has been postulated that this PDE4D5 recruitment to the agonist-activated cardiac β_2_AR poses a “brake” on the β_2_AR cAMP signaling’s ability to stimulate contractility [6,7,13,15]. By contrast, agonist-promoted dissociation of PDE from the β_1_AR underlies the more “diffuse” and more powerful at stimulating contraction signaling of this βAR subtype [6,7]. Of note, cardiac β_2_AR signaling has been reported to become more “diffuse” and decompartmentalized, i.e. to adopt a β_1_AR-like signaling pattern, in a rat model of HF, which might underlie cardiac β_2_AR dysfunction in HF [17].

On the other hand, cardiac β_2_AR can switch its signaling from G_s_ protein-mediated to G_i/o_ protein-mediated, which is believed to underlie its anti-apoptotic effects and is a feature cardiac β_1_ARs lack [18-24]. Finally, the interactions of the β_2_AR with the βarrs, which require prior receptor phosphorylation by GRKs, can have pleiotropic effects in cardiac myocytes, such as inhibition of apoptosis/promotion of survival by promoting extracellular signal-regulated kinase (ERK) signaling [25] and inhibition of inflammation by blocking the pro-inflammatory transcription factor nuclear factor-kappaB (NF-κB) [26,27], a crucial mediator of major pro-inflammatory cytokine expression, such as tumor necrosis factor-alpha (TNF-α) and interleukin-1 and −6 (IL-1 & -6) [28-30]. These βarr-dependent signaling effects may also play some part in the well known and described anti-apoptotic and other beneficial in post-MI HF effects of cardiac β_2_AR.

Cardiac GRK2 is a major negative regulator of βAR pro-contractile signaling [8-11]. By desensitizing both β_1_- and β_2_ARs, i.e. terminating their G protein-mediated signaling through cAMP, it dramatically reduces cardiac inotropic and adrenergic reserves, and since it is markedly elevated in HF, its blockade represents an attractive therapeutic strategy for heart disease treatment [8-11,31-35]. Given that GRK2 can block the pro-contractile and other beneficial signaling of cardiac β_2_AR in HF, and also that its action on β_2_AR induces recruitment of βarrs with all their aforementioned myriad effects on this receptor’s signaling, we hypothesized, in the present study, that cardiac GRK2 blockade in vivo might enhance β_2_AR signaling post-MI. In order to study the effects of GRK2 blockade specifically on this subtype’s signaling, without any interference by the qualitatively different β_1_AR signaling, we utilized the β_1_AR knockout (B1KO) mice [36], which we crossed with mice overexpressing the known GRK2 inhibitor mini-gene βARKct (or GRK2ct) specifically in cardiac myocytes [32]. After breeding the offspring to homozygosity, we studied them both under normal conditions (i.e. healthy, sham-operated animals) and after surgically induced MI to induce HF. We found that GRK2 inhibition in vivo is absolutely necessary for the β_2_AR to be capable of increasing contractility. In addition, β_2_AR anti-apoptotic signaling post-MI is augmented by βARKct, but only acutely.

## Results

### βARKct restores cardiac β_2_AR-dependent pro-contractile signaling by reducing the interaction of PDE4D with the receptor

We developed the hybrid transgenic line βARKct/B1KO by crossing the B1KO mice with the βARKct transgenic mice, which express βARKct only in cardiomyocytes. The βARKct/B1KO’s breed normally, without any gross abnormalities and present no overt cardiovascular or other phenotype (data not shown). Three-month-old male mice were chosen to undergo surgical MI in order to induce HF and were studied alongside age-matched male homozygous B1KO’s (without βARKct expression), which served as the control group.

Since GRK2 is a major (negative) regulator of cardiac βAR-dependent contractility in vivo, and the β_2_AR stimulates contractility only very weakly, we first examined the cardiac function parameters of these mice, both in sham and post-MI groups. Echocardiography revealed that the B1KO mice display significantly decreased ejection fraction compared to control wild type (WT) mice, both under normal conditions (sham groups) and at 4 weeks post-MI, as expected since the β_1_AR is the major βAR subtype in the heart stimulating contractility (Table 1) [8]. Notably, βARKct overexpression led to significant augmentation of the ejection fraction of the B1KO mice, up to the levels of WT mice, again both in normal and in 4 week post-MI mice (Table 1), while, as already known from our studies in the past [10,11,32,33], βARKct significantly augments contractility of the WT mice, as well (Table 1). Importantly, when the mice underwent in vivo cardiac catheterization to measure their hemodynamic responses to isoproterenol stimulation (a standard βAR full agonist), B1KO mice, remarkably, completely failed to show any increase in contractility (as measured by the +dP/dt_max_ LV pressure elevation parameter), even at the highest concentration of isoproterenol challenge (Max. Iso, Table 1). In contrast, the hybrid βARKct/B1KO mice showed very good contractile responses to isoproterenol, both in the sham (healthy) conditions and in post-MI HF (Table 1). As expected, the other two mouse lines, i.e. WT and βARKct, were responsive to βAR stimulation, with the HF animals in these groups showing somewhat reduced responses compared to their sham counterparts and the βARKct line displaying much more robust responses compared to the WT group (Table 1). These results strongly indicate that cardiac GRK2 is a major opposing force for the β_2_AR pro-contractile function and only when its activity is blocked (e.g. in the presence of βARKct) is the cardiac β_2_AR capable of promoting contractility in response to agonist stimulation.

**Table 1:** In vivo cardiac functional parameters

**Parameter**	**WT-Sham**	**βARKct-Sham**	**B1KO-Sham**	**βARKct/ B1KO-Sham**	**WT-MI**	**βARKct-MI**	**B1KO-MI**	**βARKct/ B1KO-MI**
**Infarct size (% LV free wall)**	N/A	N/A	N/A	N/A	41±1.2	42±2.2	40±2.6	43±3.0
**FS (%)**	41.4±1.3	48.3±2.8^	36.7±2.6^	41.3±2.4^#^	18.6±2.2	23.7±1.3^+^	11.0±1.4^+^	19.0±2.4*
**EF (%)**	72.0±1.0	78.0±2.0^	67.0±2.4^	72.0±2.0^#^	37.5±3.0	45.3±2.4^+^	23.7±2.5^+^	38.5±4.2*
**Basal HR (min–1)**	385±12	402±37	402±27	383±23	392±12	408±10	391±8	389±13
**Basal LV +dP/dt**_**max **_**(mm Hg/s)**	4692±363	6835±637^	3572±287^	4576±375^#^	3512±220	4900±172^+^	2576±155^+^	3549±231*
**Basal LV –dP/dt**_**min **_**(mm Hg/s)**	−4568±452	−6714±536^	−3269±296^	−4782±286^#^	−3515±246	−4620±203^+^	−2592±233^+^	−3471±194*
**Max. Iso-HR (min–1)**	557±36	515±40	405±28^	393±14^	509±24	581±17^+^	399±11^+^	413±20^+^
**Max. Iso-LV +dP/dt**_**max **_**(mm Hg/s)**	8825±644	13547±476^	3611±286^	9041±528^#^	5641±431	9419±601^+^	2639±139^+^	5628±419*
**Max. Iso-LV–dP/dt**_**min **_**(mm Hg/s)**	−7210±430	−8837±491^	−3199±279^	−6344±364^#^	−5880±303	−6948±277^+^	−2605±251^+^	−5548±382*

Cardiac parameter values of three-month-old wild type (WT, i.e. C57/B6), βARKct, B1KO and βARKct/B1KO mice measured at 4 weeks after MI or sham operation (Sham). *LV +dP/dt*_*max*_ maximal first derivative of LV pressure rise, *LV –dP/dt*_*min*_ minimal first derivative of LV pressure fall, *HR* heart rate, FS, fractional shortening, *EF* ejection fraction, *LV* Left ventricular, *N/A* Not applicable, Max. Iso dose: 333 ng/kg body weight. ^#^, p<0.05, vs. B1KO-Sham; *, p<0.05, vs. B1KO-MI; ^, p<0.05, vs. WT-Sham; ^+^, p<0.05, vs. WT-MI; n=7 mice/group. One-way ANOVA with Bonferroni test was performed among groups. Data are presented as mean ± SEM.

**Figure 1:**
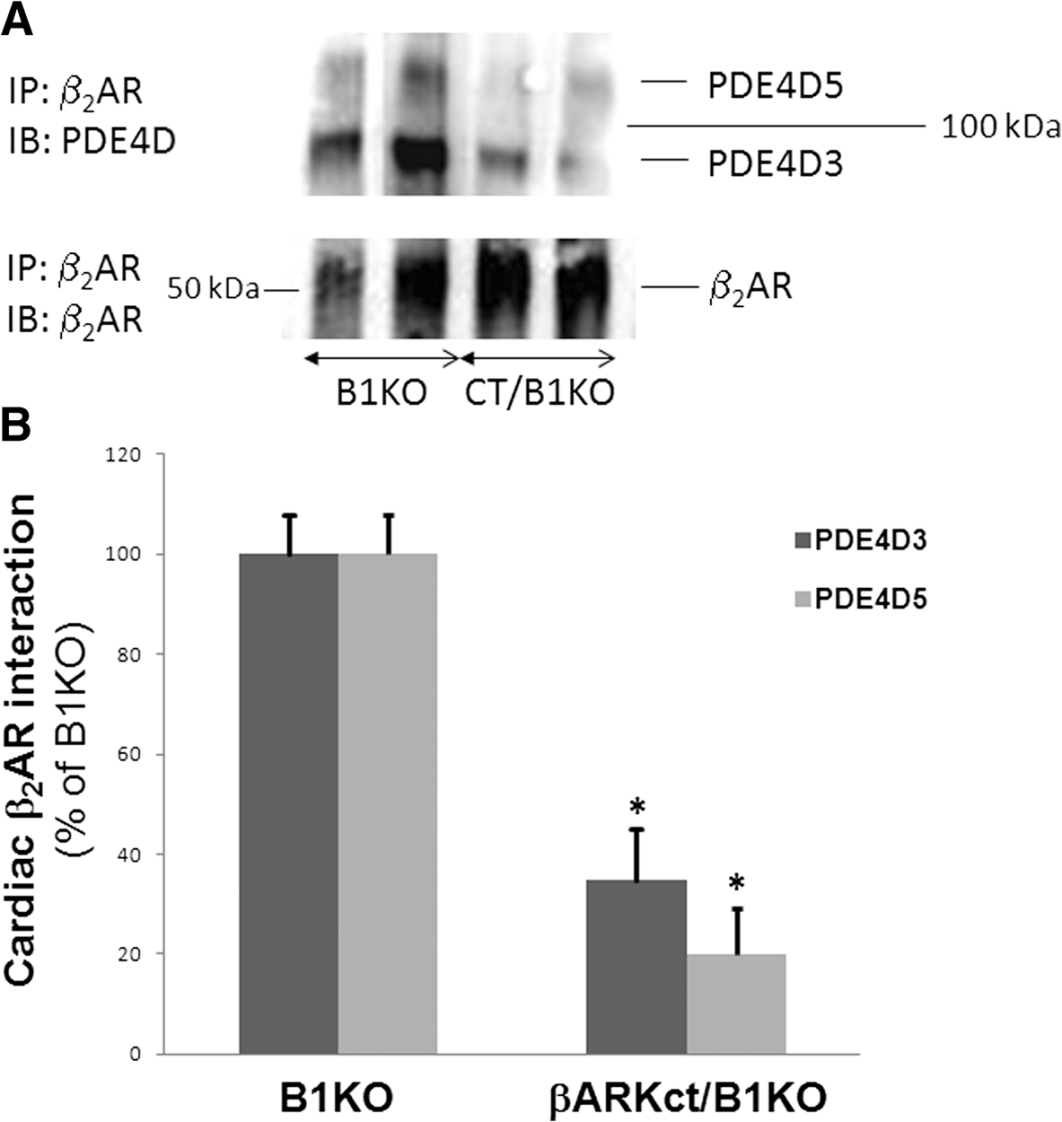
**β2AR-PDE4D interaction in the heart.** Co-immunoprecipitation (co-IP) followed by western blotting in cardiac extracts from normal (sham) B1KO and βARKct/B1KO (CT/B1KO) mice to measure the β_2_AR-PDE4D interaction in the heart. Representative immunoblots are shown in **(A)**, and the amounts of the co-immunoprecipitated PDE4D isoforms, as measured by densitometry and normalized with the amount of β_2_AR pulled down in the co-IP, are shown in **(B)**. *, p<0.05, vs. B1KO; n=4 independent experiments (i.e. performed on 4 different hearts from each mouse line). IP: Immunoprecipitation, IB: Immunoblotting.

To identify the main signaling mechanism underlying this dramatic effect of βARKct on cardiac β_2_AR-dependent contractiity, we examined the levels of PDE4D interaction with the β_2_AR in cardiac membranes of these mice in vivo. As shown in Figures 1A and 1B, the interaction of cardiac β_2_AR with both the PDE4D3 and -D5 isoforms is significantly reduced in βARKct/B1KO mouse hearts compared to control B1KO hearts, an effect that might enable βARKct to enhance cardiac β_2_AR-dependent pro-contractile signaling in vivo.

### βARKct and cardiac β_2_AR-dependent anti-apoptotic/inflammatory signaling

**Figure 2:**
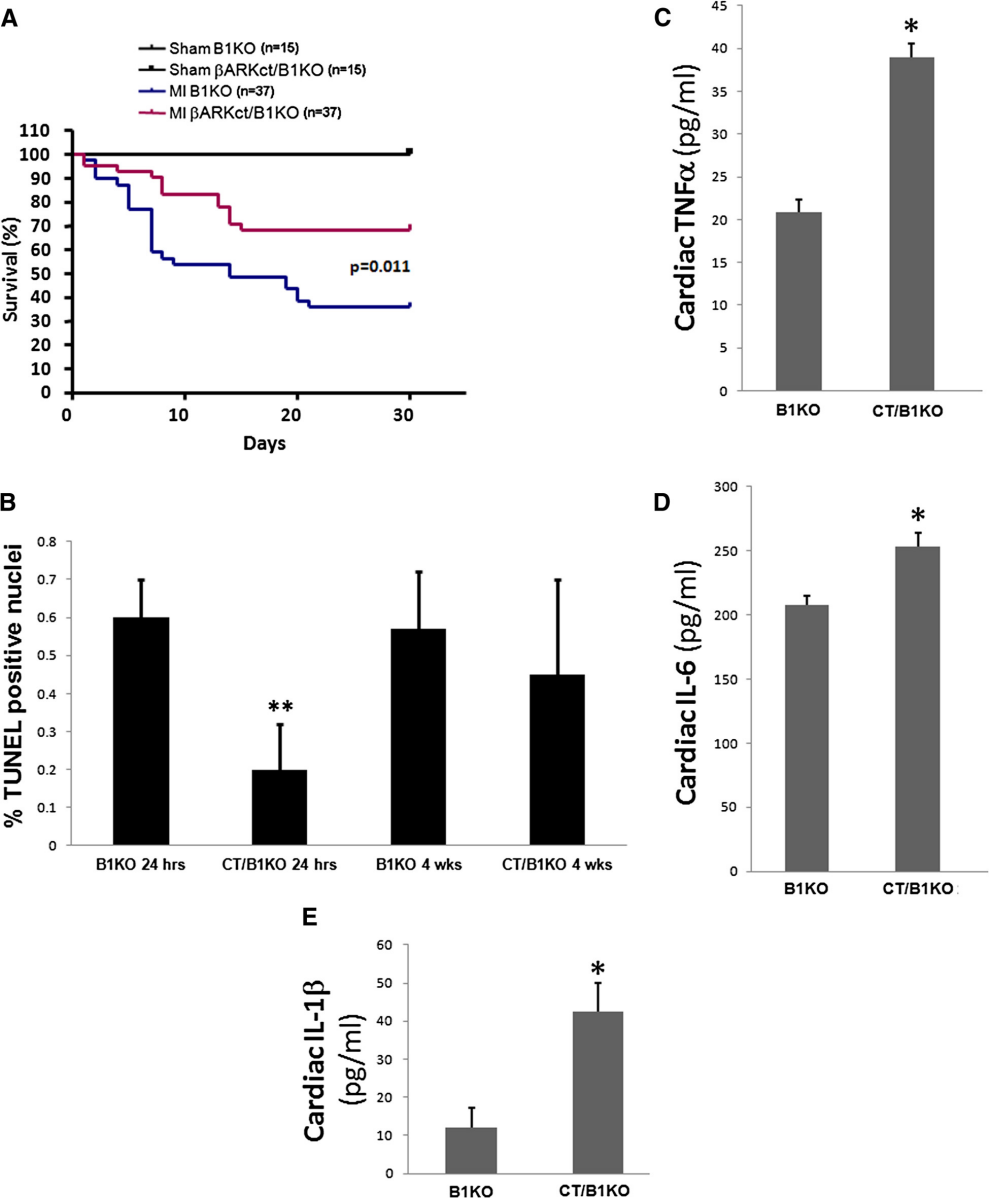
**Survival, cardiac apoptosis and inflammation post-MI. (A)** Kaplan-Meier survival curves of the 4 groups of mice of the study: sham-operated (Sham) and post-MI (MI) B1KO and βARKct/B1KO mice. p=0.011 between MI B1KO and MI βARKct/B1KO; n=15 mice/group for sham, 37 mice/group for MI mice. **(B)** Apoptotic cell death at 24 hrs and at 4 weeks (wks) post-MI in the two transgenic (B1KO and CT/B1KO) lines, as measured by TUNEL performed in the border zone of the infarct. No difference in rate of apoptosis in the remote zone at either post-MI time point was found (data not shown). **, p<0.05, vs. B1KO 24 hrs, n=6 mice/group. **(C-E)** Levels of pro-inflammatory cytokines TNFα **(C)**, IL-6 **(D)**, and IL-1β **(E)**, measured via ELISA in serum of intra-cardiac blood from B1KO and βARKct/B1KO (CT/B1KO) mice at 24 hrs post-MI. *, p<0.05, n=5 mice/group.

Next, we examined the impact of βARKct expression on the other major aspect of cardiac β_2_AR signaling, anti-apoptosis/cardiac survival. Post-MI βARKct/B1KO mice display markedly better survival post-MI compared to their B1KO counterparts (Figure 2A). Kaplan-Meier survival curves indicated that at 4 weeks post-MI, a significant (~70%) of βARKct/B1KO’s are still alive, compared to only ~40% of B1KO’s at the same time point post-MI (Figure 2A). In addition, cardiac apoptosis is found significantly decreased very early (at 24 hrs) post-MI in the βARKct/B1KO hearts compared to control B1KO hearts (Figure 2B) but similar between the two groups at 4 weeks post-MI (Figure 2B), indicating that this reduction in post-MI apoptosis induced by βARKct is short-lived. As for post-MI cardiac inflammation in the two animal groups, levels of the major pro-inflammatory cytokines TNFα(Figure 2C), IL-6 (Figure 2D) and IL-1β (Figure 2E) are significantly increased in the hearts of βARKct/B1KO mice, compared to control B1KO hearts at 24 hrs post-MI, indicating increased wound (infarct) healing-associated inflammation. By 4 weeks post-MI however, levels of all these three cytokines (TNFα, IL-6, IL-1β) in βARKct/B1KO hearts have returned to the levels of 24-hour post-MI B1KO hearts (data not shown), indicating that also the effect of βARKct on post-MI inflammation is short-lived.

**Figure 3:**
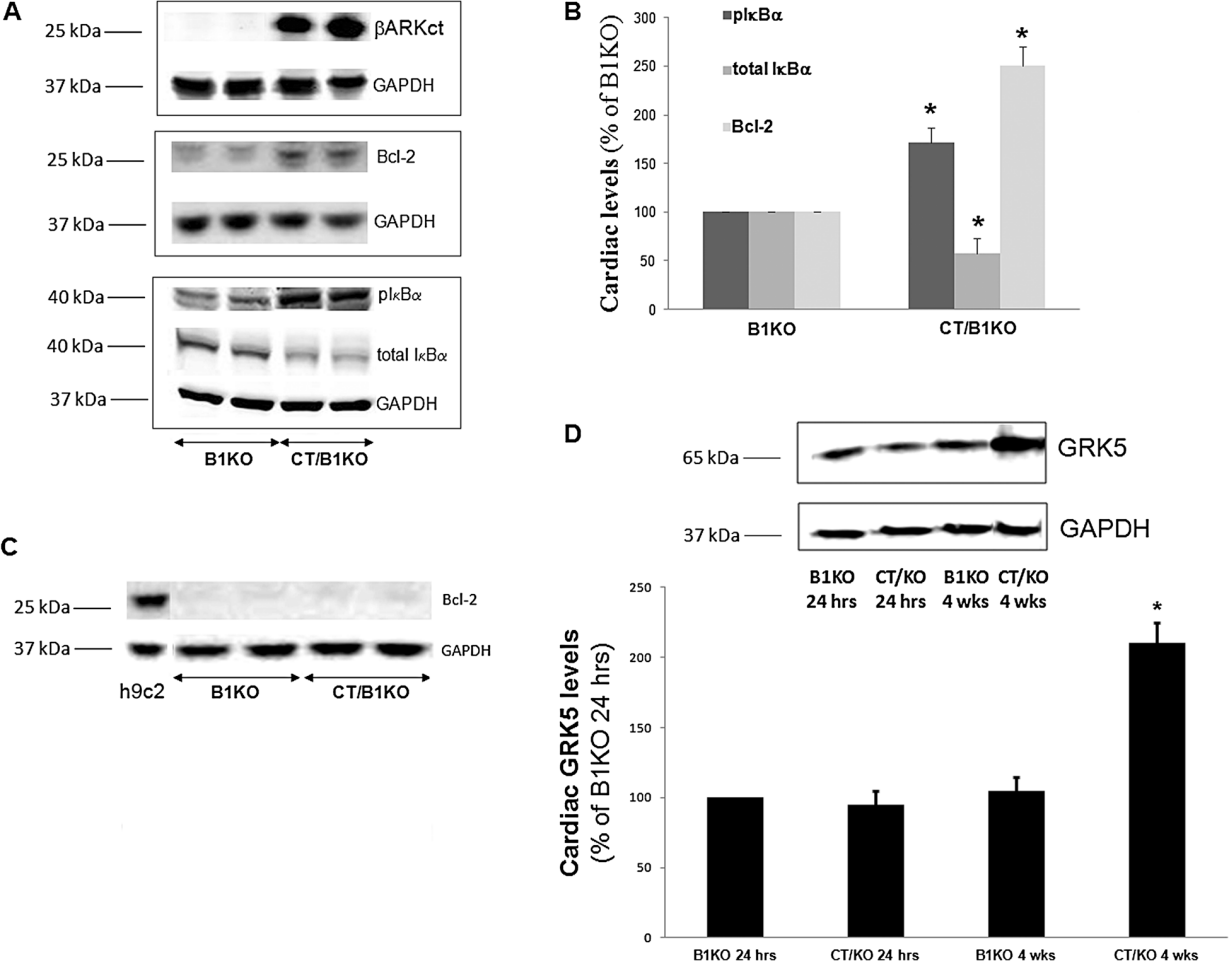
**Levels of cardiac Bcl-2, NFκB activity and GRK5 post-MI. (A-B)** Western blotting in total cardiac extracts from 24 hr post-MI B1KO and βARKct/B1KO (CT/B1KO) mice for βARKct, Bcl-2, phospho-IκBα (pIκBα), and total IκBα. Representative blots are shown in **(A)**, including blots for glyceraldehyde 3-phosphate dehydrogenase (GAPDH) as loading control for each protein tested, and densitometric quantitation, normalized with GAPDH as control and expressed as % of B1KO levels, of 4 independent experiments done in duplicate, is shown in **(B)**. *, p<0.05, vs. B1KO. **(C)** Western blotting for Bcl-2 protein in total cardiac extracts from 4 week post-MI B1KO and βARKct/B1KO (CT/B1KO) mice. Representative blots from 4 independent experiments done in duplicate are shown, including GAPDH as loading control and h9c2 cell extract as positive control (input) for Bcl-2. Bcl-2 was virtually undetectable in either group at 4 weeks post-MI. **(D)** Western blotting for GRK5 in total cardiac extracts from B1KO and βARKct/B1KO (CT/KO) mice at 24 hrs and at 4 weeks (wks) post-MI. Representative blots of 4 independent experiments done in duplicate, with GAPDH as loading control, are shown on top, and densitometric quantitation on bottom. *, p<0.05, vs. all other groups.

To identify potential signaling mechanisms underlying these effects of βARKct on apoptosis and inflammation in post-MI B1KO hearts, we examined protein levels of the major anti-apoptotic mediator Bcl-2 [37] and levels of activation of the crucial pro-inflammatory transcription factor NFκB. Bcl-2 was found significantly up-regulated in βARKct/B1KO hearts compared to control B1KO hearts at 24 hrs post-MI (Figures 3A and 3B), indicating enhanced cellular survival/inhibition of apoptosis. However, at 4 weeks post-MI, Bcl-2 protein was virtually undetectable in the hearts of both mouse lines (Figure 3C), which is consistent with the phenotypic finding of the short-lived inhibition of apoptosis in the heart by βARKct (Figure 2B). In addition, NFκB activation appears also markedly elevated in βARKct/B1KO hearts compared to control B1KO hearts at 24 hrs post-MI (Figures 3A and 3B), indicating enhanced cardiac inflammation. For NFκB to get activated, its inhibitory IκBα subunit must be phosphorylated and subsequently targeted for proteasomal degradation to release the transcriptionally active subunits of the complex [28-30]. Thus, increased phosphorylation of IκBα and decreased levels (increased degradation) of total IκBα in the hybrid transgenic hearts at 24 hrs post-MI (Figures 3A and 3B) suggest increased NFκB activation compared to B1KO hearts. Finally, blotting for βARKct in these hearts confirmed the robust expression of this GRK2 inhibitor in the hearts of βARKct/B1KO’s, which, of course, was absent from the hearts of B1KO mice (Figure 3A).

### GRK5 and cardiac β_2_AR-dependent signaling

Apart from GRK2, the other major cardiac GRK that can phosphorylate and desensitize β_2_ARs, and thus oppose β_2_AR pro-contractile and anti-apoptotic signaling, is GRK5 [8-10]. As shown in Figure 3D, cardiac post-MI GRK5 levels are initially (at 24 hrs post-MI) similar between the two groups, as is the case also in the healthy, sham-operated groups (data not shown). By 4 weeks post-MI however, a significant up-regulation (~2-fold increase) of GRK5 is observed in βARKct/B1KO hearts compared to control B1KO hearts (Figure 3D), indicating that GRK2 inhibition with βARKct leads to a compensatory up-regulation of GRK5 over time.

## Discussion

**Figure 4:**
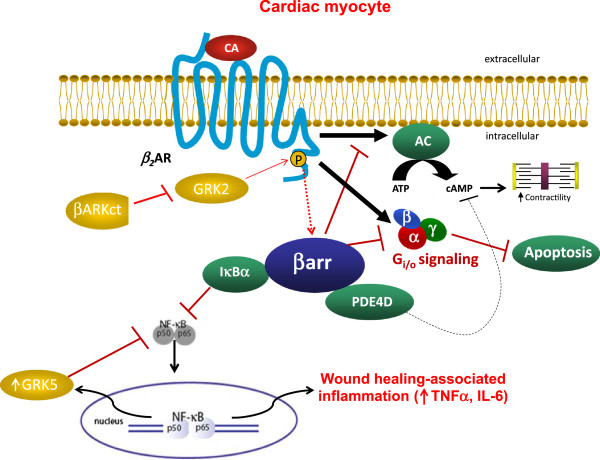
**Schematic illustration of the signaling pathways discussed in the present study that are elicited by β**_**2**_**AR activation in cardiac myocytes and are affected by GRK2 inhibition with βARKct.** CA: Catecholamine; AC: Adenylyl cyclase; ATP: adenosine triphosphate. See text for details and all other molecular acronym descriptions.

The present study reports for the first time, to our knowledge, that cardiac GRK2 is an endogenous “stumbling block” that normally prevents β_2_AR signaling from stimulating contractility, mainly because it promotes association of this βAR subtype with PDE4D in the heart, a major molecular “brake” on cardiac β_2_AR-dependent contractility [6,7,15]. Thus, only when cardiac GRK2 is blocked (e.g. with βARKct) is the β_2_AR capable of promoting cardiac contractility. Obviously, several signaling mechanisms/pathways are at play, the present study has identified the following two: 1) βARKct, by blocking GRK2, reduces the uncoupling of β_2_AR with the classical pro-contractile G_s_ protein-adenylyl cyclase-cAMP-PKA signaling pathway (Figure 4), and 2) GRK2 blockade reduces the interaction of β_2_AR with βarrs, which scaffold on themselves various isoforms of PDE4D (mainly PDE4D3 and PDE4D5) (Figure 4). PDE4D causes degradation of the local cAMP signals produced by activated β_2_AR, which are essential for stimulation of contractility, and thus it weakens these pro-contractile signals hampering β_2_AR-stimulated contractility [6,7,15]. By indirectly reducing the βarr-PDE4D interaction with the cardiac β_2_AR, βARKct releases the “brake” PDE4D poses on this receptor’s pro-contractile signaling and enhances its capacity to stimulate contractility (Figure 4).

Since the other major beneficial effect of cardiac β_2_AR signaling in vivo is inhibition of apoptosis (promotion of survival), we also tested the effects of cardiac GRK2 blockade in vivo on this aspect of β_2_AR signaling in the context of post-MI HF progression. We found that early on after MI, cardiac GRK2 blockade with βARKct also dramatically augments β_2_AR anti-apoptotic signaling, as well as its pro-infarct healing inflammatory signaling, in the heart. This results in significant reduction in all-cause mortality (marked increase in animal survival in the first 4 weeks post-MI), and reduced cellular apoptosis in the post-MI heart, compared to B1KO mice with unopposed cardiac GRK2 activity. Thus, βARKct enhances not only cardiac contractility, but also cardiac survival stimulated by the β_2_AR, which further reinforces its validity as an attractive therapeutic strategy to potentiate cardiac β_2_AR signaling and function in post-MI HF. Of course, enhancement of the anti-apoptotic signaling of other cardio-protective receptors that are also GRK2 substrates by βARKct cannot be ruled out and is, in fact, quite likely to have contributed to the observed cardiac apoptosis phenotype of βARKct/B1KO mice. However, βARKct’s cardio-protective and anti-apoptotic effects have been shown to be β_2_AR-dependent, since selective blockade of this receptor in cardiac myocytes abolishes βARKct-mediated anti-apoptotic effects [38]. On the other hand, effects of βARKct on β_2_AR-dependent pro-angiogenetic signaling, which plays an important role in peri-infarct HF progression [39], cannot be ruled out either. Nevertheless, it becomes quite clear from our current data that βARKct augments β_2_AR contractile function without negatively affecting its anti-apoptotic one, but rather actually preserving and further enhancing this β_2_AR function, as well.

However, this augmentation of anti-apoptotic signaling is short-lived: by 4 weeks post-MI, cardiac cellular apoptosis has returned to the 24-hour post-MI B1KO heart levels. This might be related to the nature of cardiac β_2_AR pro-survival signaling; β_2_AR can have remarkably different effects in the heart depending on its expression levels and on time [40,41]. Cardiac β_2_AR is known to be beneficial (i.e. promoting survival) at low levels of overexpression and in the first few months of life in mice, but when overexpressed at extremely high levels in murine hearts or later on in the mouse’s life, these animals do not survive and die of severe cardiac complications [40]. Mechanistically, cardiac β_2_AR anti-apoptotic signaling is known to proceed mainly through the G_i/o_ protein signaling pathway [20-23], to which it is capable of switching following its phosphorylation by PKA [42]. GRK2 blockade by βARKct can increase this signaling by a) decreasing the pathway’s βarr-mediated desensitization, i.e. increasing the coupling of G_i/o_ proteins with the β_2_AR, and b) by increasing the PKA-dependent switching of β_2_AR signaling from G_s_ to G_i/o_ proteins thanks to the increase of β_2_AR signaling via the G_s_ protein-cAMP-PKA (the pro-contractile) pathway it also causes, discussed above (Figure 4). With regards to the pro-inflammatory signaling of cardiac β_2_AR, βarrs are known to scaffold and stabilize the inhibitory IκBα subunit of NFκB, thereby prohibiting NFκB activation [26,27]. GRK2 blockade with βARKct decreases βarr interaction with the β_2_AR thereby “releasing” the inhibitory effect of βarrs on NFκB activation (Figure 4). Thus, NFκB activation and the subsequent pro-inflammatory cytokine production are enhanced (Figure 4). Indeed, NFκB activation and inflammatory cytokine levels were found significantly elevated in βARKct/B1KO hearts compared to B1KO hearts without GRK2 inhibition at 24 hrs post-MI.

Meanwhile however, βARKct also causes upregulation of the other major cardiac GRK, GRK5, in the first few weeks post-MI. This is also probably due to the enhanced NFκB activation (Figure 4), since NFκB can cause upregulation of GRK5 in cardiomyocytes [43]. Importantly, and given that GRK5 elevation is generally considered detrimental for the heart [8-10], this finding might explain, at least in part, the aforementioned switching of cardiac β_2_AR signaling from beneficial (anti-apoptotic) early in life of transgenic mice or at low levels of receptor expression to detrimental (pro-apoptotic) later on in the lifespan of these mice or at very high levels of cardiac β_2_AR overexpression [41]. Of note, GRK5 has also been reported to bind (via its non-catalytic, N-terminal domain) to, and stabilize IκBα, thereby inhibiting NFκB activity in several tissues, including the heart [44]. Therefore, our present findings strongly indicate that a negative feedback loop might exist in the heart, in which NFκB induces GRK5 expression, and GRK5 can subsequently suppress NFκB activation (Figure 4).

## Conclusions

In summary, the present study reports that cardiac GRK2 inhibition with βARKct in vivo is absolutely essential for the cardiac β_2_AR subtype’s pro-contractile function, all the while preserving and augmenting this receptor’s beneficial anti-apoptotic/pro-survival and pro-infarct healing signaling pathways post-MI, early on after the cardiac insult. However, the effects of βARKct on the latter signaling modalities are transient due (in part) to compensatory elevation of cardiac GRK5 over time.

## Methods

### Experimental animals and surgical procedures

The animals in this study were handled according to animal welfare regulations and protocols approved by the authors’ Institutional Review Boards. Genetically engineered, 8- to 12-wk-old β_1_AR KO (B1KO) (on C57/B6 background) [36] and the offspring of their cross with Mini-27 mice, expressing the βARKct (or GRK2ct) transgene under the alpha-myosin heavy chain gene promoter [32], were used for this study. Mice were anesthetized with a mixture of ketamine (100 mg/kg) and xylazine (2.5 mg/kg). Animals were placed in the supine position on a heated operation board and a midline cervical incision was made to expose the trachea. Following successful endotracheal intubation, the cannula was connected to a rodent ventilator. The entire left ventricle (i.e. both infarct and non-infarct zones) were used for subsequent histological and biochemical assays. Myocardial infarction was performed as previously described [35].

### Echocardiography & in vivo hemodynamics

Transthoracic echocardiography was performed with a linear 30-MHz transducer (VeVo 770 High Resolution Imaging System, VisualSonics, Toronto, ON, Canada), as described [35]. In vivo hemodynamic analysis was also performed as previously described [35].

### In situ TUNEL staining

Heart specimens were fixed with 10% neutral buffered formalin, embedded in paraffin, and sectioned at 5-μm thickness. DNA fragmentation was detected in situ in deparaffinized sections using the ApopTag Kit (Intergene) and according to manufacturer’s instructions, as described previously [45]. The total number of nuclei was determined by manual counting of DAPI-stained nuclei in six random fields per section. All terminal deoxynucleotidyl transferase-mediated dUTP nick end-labeling (TUNEL)-positive nuclei were counted in each section.

### Co-immunoprecipitation and western blotting

Cardiac extracts were prepared in 20 mM Tris pH 7.4, 137 mM NaCl, 1% Nonidet P-40, 20% glycerol, 10 mM PMSF, 1 mM Na_3_VO_4_, 10 mM NaF, 2.5 μg/ml aprotinin, and 2.5 μg/ml leupeptin. Protein concentration was then determined and equal amounts of protein per sample were loaded on SDS-PAGE gels for electrophoretic separation, as described previously [46]. For β_2_AR-PDE4D co-immunoprecipitation experiments, β_2_AR was immunoprecipitated with an anti-mouse β_2_AR antibody (sc-9042, Santa Cruz), immobilized on Protein A-sepharose beads (Invitrogen), prior to SDS-PAGE/western blotting. Total IκBα and phospho-IκBα were detected by using anti-IκBα (sc-1643, Santa Cruz) and anti-phosphoIκBα at Ser-32 (sc-7977, Santa Cruz) antibodies, Bcl-2, GRK5, GRK2/βARKct and GAPDH, with antibodies sc-492, sc-565, sc-562, and sc-25778, respectively (all from Santa Cruz), and PDE4D (various isoforms) with the PD4-401AP antibody (FabGennix). Immunoblots were revealed by enhanced chemiluminescence (ECL, Amersham Biosciences) and visualized in the FluorChem E Digital Darkroom (Cell Biosciences). Densitometry was performed with the AlphaView software (Cell Biosciences) in the linear range of signal detection (on non-saturated bands).

### Cytokine measurements via ELISA

Pro-inflammatory cytokines TNFα, IL-6 and IL-1β were measured in serum obtained from left ventricular blood, immediately prior to heart excision and animal euthanizing, via multiplexed ELISA, as described [47,48]. The assay was performed using the Mouse Cytokine ELISA Profiling Kit (EA-1091, Signosis), according to the manufacturer’s instructions.

### Statistical analyses

Data are generally expressed as mean ± SEM. Unpaired 2-tailed Student’s *t* test and one- or two-way ANOVA with Bonferroni test were generally performed for statistical comparisons, unless otherwise indicated. For all tests, a p value of <0.05 was generally considered to be significant.

## Abbreviations

βAR: Beta-adrenergic receptor; B1KO: β_1_AR knockout; GRK: G protein-coupled receptor kinase; βARKct: Beta-adrenergic receptor kinase (GRK2) carboxyl terminal fragment; βarr: Beta-arrestin; PDE: Phosphodiesterase; MI: Myocardial infarction; HF: Heart failure; NFκB: Nuclear factor-kappaB; IκBα: Inhibitor of nuclear factor-kappaB alpha subunit; cAMP: 3′-5′ adenosine monophosphate (cyclic adenosine monophosphate); WT: Wild type; PKA: Protein kinase A; Gs: Stimulatory G protein; Gi/o: Inhibitory or other G protein; TNFα: Tumor necrosis factor alpha; IL: Interleukin; TUNEL: Terminal deoxynucleotidyl transferase-mediated dUTP nick-end labeling; Bcl-2: B-cell lymphoma 2; LV: Left ventricular; ELISA: Enzyme-linked immunosorbent assay.

## Competing interests

The authors declare that they have no competing interests.

## Authors’ contributions

NCS, XV, AS, GR, AC, DLic, CDL, and EG performed research. DLeos assisted with writing of the paper. WJK supervised the project, provided funding for the research and assisted with writing of the paper. AL conceived and supervised the project, designed research, provided funding for it, and wrote the paper. All authors have read and approved the final manuscript.
